# Harm reduction interventions in HIV care: a qualitative exploration of patient and provider perspectives

**DOI:** 10.7717/peerj.1932

**Published:** 2016-04-14

**Authors:** Suzanne Carlberg-Racich

**Affiliations:** 1 Master of Public Health Program, DePaul University, Chicago, IL, United States

**Keywords:** Harm reduction, HIV care, Injection drug users, Qualitative research

## Abstract

**Background.** A culture of stringent drug policy, one-size-fits-all treatment approaches, and drug-related stigma has clouded clinical HIV practice in the United States. The result is a series of missed opportunities in the HIV care environment. An approach which may address the broken relationship between patient and provider is harm reduction—which removes judgment and operates at the patient’s stage of readiness. Harm reduction is not a routine part of care; rather, it exists outside clinic walls, exacerbating the divide between compassionate, stigma-free services and the medical system.

**Methods.** Qualitative, phenomenological, semi-structured, individual interviews with patients and providers were conducted in three publicly-funded clinics in Chicago, located in areas of high HIV prevalence and drug use and serving African-American patients (*N* = 38). A deductive thematic analysis guided the process, including: the creation of an index code list, transcription and verification of interviews, manual coding, notation of emerging themes and refinement of code definitions, two more rounds of coding within AtlasTi, calculation of Cohen’s Kappa for interrater reliability, queries of major codes and analysis of additional common themes.

**Results.** Thematic analysis of findings indicated that the majority of patients felt receptive to harm reduction interventions (safer injection counseling, safer stimulant use counseling, overdose prevention information, supply provision) from their provider, and expressed anticipated gratitude for harm reduction information and/or supplies within the HIV care visit, although some were reluctant to talk openly about their drug use. Provider results were mixed, with more receptivity reported by advanced practice nurses, and more barriers cited by physicians. Notable barriers included: role-perceptions, limited time, inadequate training, and the patients themselves.

**Discussion.** Patients are willing to receive harm reduction interventions from their HIV care providers, while provider receptiveness is mixed. The findings reveal critical implications for diffusion of harm reduction into HIV care, including the need to address cited barriers for both patients and providers to ensure feasibility of implementation. Strategies to address these barriers are discussed, and recommendations for further research are also shared.

## Introduction

Harm Reduction efforts aim to reduce the harmful effects of drug use (related to health, social relationships or economics) on the PWID, the community and society as a whole (Newcombe , 1992). Implicit in this definition is the establishment of a respectful, nonjudgmental and client-centered relationship, which works with the individual to define incremental and manageable steps toward better health. This means that counseling to reduce drug-related harm is tailored to an individual’s needs, and that they are trusted to choose options that work for their own positive change. Despite decades of research documenting the effectiveness of harm reduction approaches, particularly needle and syringe exchange programs (NSPs). Over the years, NSPs have been rigorously studied and shown to be effective at reducing injection-related risk behaviors (Heimer, 1998; Hagan et al., 1995), HIV transmission (DesJarlais et al., 2009; DesJarlais et al., 1998), Hepatitis transmission (DesJarlais et al., 2005) and facilitating entry into treatment (Brooner et al., 1998; Hagan et al., 2000), yet the need for safer injection supplies remains unmet, and barriers to providing harm reduction services persist. Globally, persons who inject drugs (PWIDs) receive less than two clean needles per month, hardly enough to meet individual needs to reduce harm and to curb the dramatic spread of HIV and Hepatitis (Mathers et al., 2010), and far less than the minimum of 200 per annum/per PWID recommended by The World Health Organization (Zandonella, 2006). While some coverage may exist in the US, distribution rates are also low when compared to WHO NSP goals, with approximately 23 needles/syringes distributed annually per PWID (Harm Reduction International, 2012), and disproportionate access for urban residents versus those living in suburban or rural areas (Des Jarlais et al., 2015).

The availability of sterile syringes alone is not sufficient to prevent HIV and Hepatitis transmission, rather, risk reduction counseling or education should also be provided to guide PWIDs regarding the sharing of other equipment in the process. The sharing of cookers and cotton filters may exist even among PWIDs who use sterile syringes (Hagan et al., 2001), suggesting that provision of sterile works along with education and/or safer injection counseling may be necessary to curb infection rates, yet these services can be difficult to provide in chronically underfunded US NSPs. Counseling and the full range of sterile injection supplies are not always available within the scope of US based harm reduction services. Harm reduction counseling around other drug use is also needed, since smokers of crack cocaine are also at elevated risks for HIV (DeBeck et al., 2009), and harm reduction can assist in reducing that risk. Alternative venues for such services might help to fill the critical gaps, such as clinical care environments, drug treatment facilities, and correctional systems. One place to start may be the HIV care environment, where efforts to incorporate other harm reduction approaches exist, even if on a very limited level. For example, the HIV care clinic has been a target for expansion of opioid substitution therapy, particularly with the use of suboxone. Within a clinical care environment, the adoption of a harm reduction philosophy of non-judgement, along with a specific set of interventions (safer injection counseling, safer stimulant use counseling, overdose prevention, equipment distribution) would offer an additional venue to meet current need. However, little is known about whether patients or providers would be receptive to these opportunities.

### The cultural, political and fiscal context

In the US, the dearth in services can be explained by the philosophical, legal and funding barriers to providing syringe exchange. For example, a ban has prohibited the use of federal funding for NSPs for several decades, relegating most NSPs to acquire funding through other means. Even recent changes to this legislation through the congressional Omnibus that allow funding for other services within NSPs, while helpful, still prohibit the use of funding for the actual syringes. The lack of a comprehensive harm reduction approach can perhaps be best explained by the dominant cultural paradigm in the United States as it relates to substance use, which assumes that those who use substances have no control over their use, responds to patient use in a shame-based manner, and assumes they are either in recovery or relapse—rather than recognizing a vast area between those two stages (Fitzgerald, 1996). In particular, the perception that drug use and addiction are moral problems perpetuates punishing approaches to drug use rather than facilitating accessibility to treatment. For example, in 2012, drug charges accounted for over 50% the federal prison population, with 80% of those due to possession only (Drug Policy Alliance, 2014).

In the US, drug-related stigma has vast implications. Discrimination is associated with poorer health—both physical and mental, and alienation is associated with poor mental health (Ahern, Stuber & Galea, 2007). Considerable negative stereotypes associated with being a “drug user” persist, and serve to delegitimize persons who use drugs, which is further compounded with the stigma of being HIV positive (Ware, Wyatt & Tugenberg, 2005). Stigma may also result in engagement in riskier behavior, and interfere with a drug user’s willingness to use harm reduction services in the community (such as syringe exchange services) due to fear or embarrassment (Simmonds & Coomber, 2009), making the need to access harm reduction services in other venues even more urgent. While harm reduction services are often a bridge to healthcare or treatment, they rarely exist within healthcare environments in the US. Rather, syringe provision has occurred in clinical care on a prescription program basis (Rich et al., 2001), but even that approach has been limited. Stigma within the HIV care system is also problematic. For example, PWIDs are often presumed to be incapable of adherence and subsequently denied access to life-saving antiretroviral therapy (ART), despite evidence that PWIDs can be adherent, particularly when opioid substitution therapy is provided (Wolfe, Carrieri & Shepard, 2010; Lert & Kazatchkine, 2007). Despite the availability of buprenorphine for office-based medical management of opioid addiction, 96% of states do not have the treatment capacity to properly address their rates of dependence (Jones et al., 2015). Problems of access are further compounded by racial disparities, as African-Americans are less likely to access substance abuse treatment, and typically receive lower doses of methadone in treatment than Caucasians (Rosen, 2004). This is particularly problematic considering the disproportionate rates of HIV and drug use in African-Americans, who also experience more problems related to their drug use than other racial groups (NIDA, 2003).

Chicago’s climate is similar, particularly as it relates to racial disparities. For example, arrests for possession have historically demonstrated who is most affected by drug policies. African-American males are arrested for possession of marijuana at fifteen times the rate of Caucasian males within Chicago (Substance Abuse and Mental Health Services Administration, 2013). Inside Cook County (which encompasses both Chicago and some surrounding suburbs), African Americans are more than twice as likely to be prosecuted for drug crimes than Whites, and also suffer sentencing disparities (Lyons et al., 2013).

With limited funding, harm reduction services are still delivered through a few programs in Chicago, most notably the Chicago Recovery Alliance, and Community Outreach Intervention Projects, who provide fixed-site and outreach based services to PWIDs and other people who use drugs in Chicago. However, funding constraints limit opportunities for expansion to meet current need, particularly into additional suburban and rural areas. Thus, more options are necessary to ensure safer injection and reduction of harm among Chicago IDUs.

An untapped opportunity for expansion within the city exists in HIV care clinics, where PWIDs are accessible and clinicians are expected to engage in some level of prevention work with patients. In 2003, the Centers for Disease Control and prevention (CDC) began to focus on the need for prevention in the HIV care environment. HIV care clinicians were ideal targets for training as they already had daily access to those infected with HIV. A few nationally diffused interventions were shown to produce patient-level behavior change when counseling was initiated by the clinician (Richardson et al., 2004, for example), but measured outcomes were related to reducing risks from sexual exposures. In available evidence-based curricula, messages regarding drug use have mainly focused on: (1) abstaining from drug use or, (2) using clean needles, messages that do not meet the needs of all PWIDs or those using stimulants like crack cocaine. More options for messaging and interventions would better meet the needs of HIV-positive patients in the city, if the patients and providers are willing to participate.

The integration of particular interventions in HIV care may be lifesaving for those who are not accessing it elsewhere. In particular, a harm reduction (client-centered, nonjudgmental) approach may enhance the level of trust between provider and patient, and allow space for specific interventions: safer injection counseling, safer stimulant use counseling, overdose prevention, and syringe/needle exchange or distribution. These interventions can provide the means to prevent additional viral infections (HCV, for example) or other complications, such as abscesses, cellulitis, endocarditis, necrotizing fasciitis, etc. These infections occur at various points during the injection process, from the sharing of syringes, to the sharing of paraphernalia (cookers, cotton filters, water, etc.), to the reuse of one’s own injection equipment, to the splitting of drugs between users in liquid form (Roman-Crossland, Forrester & Zaniewski, 2004). Although the sharing of syringes is a well-known risk, the harms related to the sharing of other equipment may not be well understood by the clinician, or the patient. The entire injection process can vary from user to user, as can the equipment. Only by exploring each individual user’s process can clinicians assist in offering options that reduce harm associated with a given injection. With proper instruction, clinicians may be able to offer a multitude of harm reduction interventions, and assist the patient in identifying steps toward healthier use which are realistic to the patient, with an approach based on trust and acceptance, rather than stigma and judgment. However, little is known about how providers or patients would feel about having a harm reduction approach and interventions within the HIV care visit, a gap in the literature this study began to explore.

## Materials & Methods

This study intended to capture and understand the points of view of both patients and providers, efforts which were facilitated by a qualitative, phenomenological paradigm that imbued the research with in-depth perspectives and experiences. Both patients and providers were interviewed, as counseling interventions specifically require willing participants on both ends. Having both perspectives adds to the depth of understanding of the care visit, and allows for triangulation of data in understanding the phenomenon under study. Research Questions were drafted accordingly and guided the process (see Table 1).

**Table 1:** Research questions.

Line of inquiry	Specific research questions
Attitudes regarding harm reduction counseling	∙ How do HIV care providers feel about engaging their active substance-using patients in harm reduction counseling?
∙ What barriers (if any) exist to incorporating such services into the HIV care visit?
∙ How do active substance using patients (living with HIV or Hepatitis) feel about discussing harm reduction concepts with their care providers?
Implications for practice	∙ What do the findings of this study tell us about the feasibility of incorporating harm reduction into clinical care?
∙ What might facilitate the diffusion of harm reduction into clinical care, from the provider and patient perspective?

To address these questions, semi-structured interview instruments were drafted, pilot tested with the target audiences, and revised. This type of instrument allows both focus and flexibility for emerging topics as they occur within an interview. Paired with the semi-structured interview guide was a conceptual map of inquiry that depicted key domains and topics to pursue—a visual guide to data collection. Questions and domains were created based on the research questions as well as familiarity to participants. For example, in order to ascertain Harm Reduction knowledge and attitudes regarding practice, case-based questions were crafted which asked the clinician to consider how they might approach a situation. To augment the case-based questions and encourage rich attention to detail, props were also incorporated into the interviews. For both the clinician and patient interviews, safer injection equipment, safer crack smoking equipment, and overdose prevention materials were placed on the table and referred to during specific interview questions. Language on the patient instrument was evaluated extensively in the pilot phase, with member checking to avoid jargon, and to make the process more conversational in nature.

### Selection and description of participants

To focus on those who suffer the greatest impact related to their drug use in Chicago, a purposeful sample was selected from three Ryan White-funded clinics located in areas of Chicago with a high prevalence of HIV and drug use, in underserved, low-income African-American communities. The sample was designed to be ‘information rich’ (Patton, 2002) in its ability to yield insight into this group of patients’ perspectives on harm reduction interventions in the care visit. Inclusion criteria for the patient sample included: being 18 years or older, able to speak English, having HIV, Hepatitis B or Hepatitis C infection, and actively using either heroin or crack cocaine (based on a short screening instrument). It was understood that all patients would be living with HIV infection given their care within the HIV clinic. Patients were recruited by placing flyers in each clinic that included the purpose of the study, the desired target population, and the IRB protocol number. In addition, clinics referred patients who met inclusion criteria. All subjects were offered the opportunity to be interviewed on-site at the clinic, or at another location. Patients were compensated $20.00 for their time, with careful consideration to determine a modest amount which would not coerce patients to participate clinicians were also a purposeful sample, with the aim of interviewing all prescribing clinicians in each of the selected clinics. Inclusion criteria for clinicians included: being 18 years of age or older, able to speak English, and currently providing services (including prescribing medications) to HIV/AIDS patients who actively use drugs. Clinicians were recruited with letters explaining the purpose of the study and IRB protocol information, and follow-up calls were made to encourage participation and facilitate scheduling. Clinicians were compensated for their time with a combination of training vouchers and a resource package containing tools and guidelines to assist the clinician in treating HIV, Hepatitis and addiction. The study was approved for human subjects’ research by the Institutional Review Board (IRB) at the University of Illinois—Chicago, Protocol #2007-0116.

### Data collection, analysis, and assurance of rigor

The majority of interviews were conducted within the clinics in a private space. In two cases, patients chose an alternate location to preserve their privacy. Written informed consent was received from all eligible subjects, and all agreed to be audio-taped for transcription. Audio recordings were transcribed by a professional transcription service and then verified for accuracy. All transcripts were entered into ATLAS.ti, a software program that aids the analysis and management of qualitative data (Muhr & Friese, 2004).

A deductive analysis approach was used to generate themes, since the goal was to explore pre-determined concepts from the perspectives of the participants (Patton, 2015). Thus, the first step in the analysis was the creation of an index code list for both the clinicians and the patients, based on the research questions, interview guides, and concept maps. These broad categorical codes and their definitions were used in the initial process of open coding. Initial coding occurred while interviews were ongoing. Emerging, unanticipated themes were used to create additional codes for analysis, and relationships among codes were identified (a process referred to as axial coding) resulting in more refinement of the code list, with changes in codes recorded by the researcher for transparency in process. During the process, the data was coded three times. The first round was done manually, printing all transcripts and assigning codes in the margins, and noting any emergent themes. The second round was done within ATLAS.ti, without making reference to the first round to ensure fresh thinking about the data. At this point, codes were refined again, and a third round ensued, comparing the previous rounds and utilizing the constant comparative method to ensure fidelity to the code definitions. Additionally, an experienced independent qualitative researcher coded a subset of interviews to assess the thoroughness of code definitions and to mitigate any researcher bias. The Principal Investigator had experience in harm reduction, and the external coder did not—allowing for fresh thinking about the data and code definitions. Inter-rater reliability was calculated with Cohen’s Kappa which accounts for concordant coding that might occur by chance (Cohen, 1960), making it a more robust measure of inter-rater reliability. Kappa results were interpreted with the use of guidelines published by Landis & Koch (1977), which provide a benchmark for discussion around discordant coding.

To assure scientific rigor within a qualitative, constructionist paradigm, researchers must establish and enhance trustworthiness (Lincoln & Guba, 1985). This study employed strategies to enhance all four components of trustworthiness: credibility, transferability, confirmability and dependability. Participants were engaged in member-checking, the technique that is most critical in establishing credibility (Lincoln & Guba, 1985), by ensuring that notes taken during the interview were accurate representations of the realities shared by the participants, both in their individual contributions and to vet emerging themes in the data. This was done throughout the interview as needed and as a final step in the interview process, summarizing both emerging themes and specific passages in the notes to ensure fidelity to participant experience. Peer debriefing was also used, by engaging in a purposeful discussion with peers who work in HIV care environments (a former PWID and a former non-injection drug user) during instrument development, and to verify emerging themes. These peers were able to provide insight and feedback on interpretation of study data by the researcher to identify any potential bias (see Lincoln & Guba, 1985). Triangulation of data sources was used in the data collection process by inquiring about the same concepts from differing perspectives (Patton, 2015), applied here as both patient and provider views on the same issues. Similarly, it was used in data analysis by employing an independent coder to code a subset of interviews for the purposes of comparing findings and calculating the IRR, called “triangulating analysts” (Silverman & Marvasti, 2008). Although the results here cannot be broadly generalized, they may be transferable to clinics in urban areas that serve similar populations (see the following sections for details: ‘The Cultural, Political and Fiscal Context’, the ‘Selection and Description of Participants,’ as well as ‘Sample Demographics’). As such, comprehensive descriptions of the urban setting and clinic populations may assist in determining transferability to similar settings. In addition, thick description was achieved with detailed notetaking related to participants, clinic context/interactions, and perceived relationships, in addition to the evolution in the coding process. When combined with raw data (transcripts and audio files) such materials become an audit trail of the research, to facilitate replication of the study methods to verify results. This audit trail works to ensure the “confirmability” and the “dependability” of the results generated in this research (Lincoln & Guba, 1985).

## Results

### Sample Demographics

A total of 32 patient participants were screened and *N* = 31 were determined to be eligible based on the inclusion criteria. All 31 eligible subjects consented to the process, agreed to be audio taped, and participated in the interviews (*N* = 31). All participants were African-American. The gender breakdown was intentionally skewed towards males to approximately match the Chicago PWID population (G Scott, N Prachand & C Ciesielski, 2005, unpublished data) with 8 female participants (26%) and 23 male participants (74%). Although not asked specifically about sexual orientation or gender of sex partners, a portion of male subjects reported having sex with other men (*n* = 9 or 39% of the males). Approximately half of the participants (*n* = 15 or 48%) reported injection drug use of heroin, while the other half (*n* = 16 or 52%) were primarily crack cocaine users with some reports of injection. Most reported polydrug use, particularly the use of alcohol or marijuana in addition to their drug of choice, or the use of both heroin and crack cocaine (*n* = 8, or approximately 25% of subjects). Most participants were experienced drug users with long careers of using, a high risk group for opioid overdose. See Table 2 for the detailed breakdown by age, and Table 3 for the breakdown by level of education.

**Table 2:** Age range of participants (*N* = 31).

Age range	Number of participants	Percentage
20–29	1	3%
30–39	3	10%
40–49	13	42%
50–59	13	42%
60–69	1	3%
Total	*N* = 31	100%

**Table 3:** Educational level of participants (*N* = 31).

Educational level	Number of participants	Percentage
Less than high school degree	8	26%
High school graduate/GED	7	23%
Some college	10	32%
College graduate	2	6%
Trade school	4	13%
Total	*N* = 31	100%

As previously mentioned, all clinics were publicly-funded (Ryan White, primarily), and situated in areas of high HIV prevalence and heroin/crack use, in low-income areas of the City of Chicago. All clinics served mainly African-American patients. All three included additional services, including mental health counseling, Ryan White Case Management, and counseling for substance use disorders. All three also had peer group support services available. At each clinic, the physician in charge of HIV care was interviewed, as well as at least one nurse practitioner who provided medical care to the target population, for a total of seven (*N* = 7) interviews. Although this sample is small, it represented 100% of the HIV care clinicians at the three participating clinics, as each clinic had only one physician providing HIV care and one or two nurse practitioners. The majority of clinicians (five out of seven) had been working in HIV for well over ten years, while one other clinician had been working in HIV for over five years, and another more than two years. Most of the clinicians had very limited experience working with active substance users before beginning their work in HIV. All four of the nurse practitioners were female, and two of the three physicians were male. Four of the providers were African-American (two physicians and two nurse practitioners) and three were Caucasian (one physician and two nurse practitioners).

### Patient knowledge of harm reduction

Before asking patients how they would feel about incorporating harm reduction into the HIV care visit, they were asked about their own knowledge of harm reduction, and whether they had been participants of any harm reduction programs or trainings. If they had not received any information about harm reduction from programs or trainings, they were asked what they did to reduce harm related to their use, and how they learned to do those things. Participant responses varied, as did their perceptions of the reduction in harm, encompassing everything from avoiding harmful products to avoiding financial harm as a result of their use. The majority of participants learned how to reduce harm as a result of their use by listening to or watching other users, rather than via harm reduction programs. As one participant explains,

You know, people talk about communities and in every community, even those of us that use, it’s someone be able to tell you a safer way to do what you’re doing.

Participants gained valuable knowledge from other users, such as the importance of buying from trusted sources, and using a glass rather than a metal crack pipe. For example, one participant explained:

Well, they would say don’t go over here and buy from this place because they’re not selling real stuff, you know. Their stuff is mixed with something that they don’t know what it is. They had something that they got from them that made them sick. Or they would say, like, they don’t use metal pipes and stuff because you scrape that stuff and the metal get into your system.

Still others cited learning by watching others such as this comment of how a participant decided to begin using a mouthpiece,

I guess from the experience of seeing sores on other people’s mouths.

Other participants were unable to articulate any information learned about reducing harm or recall any sources for information, such as the comment by this participant,

Nobody never talked to me about that. I never knew there were no safety issues in that.

A smaller group of participants cited that they learned how to reduce harm through ‘common sense,’ such as in the following comments:

I don’t want to suck on nothing nobody else been sucking on and spitting all in—it’s just common sense.,

or relied on themselves for information such as this participant,

I teach myself. Through my experiences, you know, word of mouth, and my own knowledge of common sense.

Similarly, some participants reported learning ‘the hard way,’ through experience, such as in the following quotes:

That’s how I learnt not to spend my money and make excuses or taking my money buying drugs, trying to trick myself, well I’m going to get this money next week and I’m going to replace it. There ain’t no such animal…it never got replaced and then I was deeper and deeper in debt

I have learned to pay rent and bills first, and them if I spend whatever’s left, okay, fine, everything’s taken care of. Trial and error. Screwing up.

Of particular interest, only two participants cited harm reduction programs as a learning source for this type of information, and were currently using an exchange for their source of syringes. For safer injection equipment, most participants utilized multiple sources. For example, one patient said,

Well, I have gone to a syringe exchange and I have bought them in the projects.

In fact, almost a third of injection drug using patients had also purchased their syringes on the street at some point, such as this participant,

They sell them on the street. A bag of them for five dollars, a bag of ten for five dollars, or one for one dollar.

Another third of patients had purchased their syringes in the pharmacy or drugstore, like this subject,

We’d go to the drugstore, buy some, we all shared. We could always go buy some, but you know, we didn’t know about that. You bleach it, you run bleach through it, then rinse it with some water. That’s all we thought we ever needed.

Similarly, almost a third of patients reported stealing their syringes from diabetics, usually who were a part of their families. One patient explains,

My mother was a diabetic…. She would leave them. She didn’t keep them for me. She didn’t know she was…yeah, she’d keep them in her empty box container, the red one, and throw them away, but I’d wait and use them.

In this study, very few participants were currently connected to syringe exchange or harm reduction programs, despite their relative geographic proximity to local Chicago mobile sites. In addition, only one provider mentioned making regular referrals to syringe exchange programs. Given the dearth of patient engagement in external harm reduction programs and the lack of provider referrals, it is clear that a gap exists for these patients, even with their own efforts to reduce a range of harms related to their use. It would therefore be helpful to know whether HIV care would be a suitable alternative environment to provide harm reduction interventions to fill this gap. A discussion of patient and provider themes that emerged from the inquiry around its potential are discussed below.

### Patient themes

#### “With open arms”

Patients (27 out of 31, or 87%) were very open to receiving safer injection/safer crack smoking counseling from their clinicians. Receptiveness was only part of the response, as the majority reported that they would feel grateful for such relevant information. Patient rationale for this openness included gratitude for information that might assist participants in maintaining or improving their own health, or that might convey a provider’s concern about their patient. For example, participants said:

I would open up and accept that with open arms, because information is powerful. I mean, if there’s anything I’m doing that shouldn’t be done, or you have solutions or you showing me some concept, I’m gonna accept it. (Female PWID)

I would feel comfortable if I was injecting and they told me a safer way of doing it instead of just in the alley, you know what I’m sayin.? (Male PWID)

#### Approach matters

A small subset of participants (*n* = 4, or 13%) expressed diverging opinions about the concept of harm reduction counseling in the visit. These diverging opinions are critical to address, and seemed most situated in an abrupt change in approach as an issue, after having their providers tell them to quit over a substantial period of time. They explained that they would be surprised by this conversation coming from a health care provider. Nonetheless, they also anticipated responding with openness and gratitude for harm reduction counseling in the visit. For example: one such participant said,

Because all the time that I’ve known him, he’s encouraged me to stop using. But then I would be slightly surprised, because I would take that information that can’t hurt since you’re not gonna stop, do it this way, which shows concern once again. (Male crack user)

For this participant, the change in approach might be surprising initially, but eventually seems to represent provider-level concern for the patient’s health. Similarly, one patient talked about this conversation representing a shift from telling him that drug use is bad, to saying that it is okay. He explained:

I mean it’s a good thing that she explains it to me, but that’s like letting me know it’s okay to do heroin from a doctor’s point of view. It may sound crazy but for your doctor to tell you, you can use it but don’t use it like this cause you’ll OD., that’s like letting you know it’s okay. (Male PWID)

Interestingly, after the interviewer rephrased the question and said, “What if she asked you whether you were ready to go into a program, and you weren’t ready to go, and she said, ‘if you’re not ready to go into a program, let’s talk about some of the ways that you can stay safe.’ What would you think?” With this change, the participant said, “I’d be willing to hear it” and explained that it was all about the choice of words. Thus, if clinicians are trained to engage patients in safer injection counseling, the conversational approach becomes quite important in sending a message that the clinician is still concerned about the patient when such abrupt changes in the usual messages are provided.

#### “It’s a good place to start”

When asked how they would react to their clinician providing safer injection supplies (new syringes, cookers, filters, etc.) or safer crack smoking kits (mouthpieces, screens, chap stick, etc.), all but two (*n* = 29, or 94%) participants expressed that such a program would be helpful. Participants remarked,

It’s a good place to start because many times I’ve come here and didn’t have a syringe and I’ve used somebody else’s because I didn’t have $1.00 to buy some, so this would be a good place to start. (Female PWID)

What better ways to get it and be safe than to get it from a safe person? (Male PWID)

You know, he’s just trying to look out for me. You know, I don’t want you on. I’m not saying, it’s good for you to do drugs. I don’t want you to do drugs, but if you’re gonna do drugs, this is the safest way. (Female crack user)

However, again there were a few participants who felt that providing such a kit would not be helpful. For example, one participant said,

But I don’t think they will do that for us people that smoke crack. It’s no safer way to smoke crack because one way or another, either if you’re smoking out of an antenna, you’re gonna burn your lip, you’re gonna burn your fingers. (Male crack user)

This participant did not report engagement with a local Harm Reduction program which may have shared ways to avoid these types of burns, suggesting a need for more focus on crack in local Harm Reduction efforts. While this less-receptive perspective was shared by only a small fraction of patients, it belies an importance on training clinicians to respond to each patient according to their readiness to receive the information. However, lack of such training was often found to be a significant barrier from the perception of providers.

Despite patient-expressed openness to the idea of receiving various harm reduction interventions within HIV care, their willingness to be candid about their current use varied. Fifteen participants (48%) felt very comfortable discussing their use and understood the need to be open with medical providers or their physician. For example, one subject shared her general comfort in discussing her use, but was somewhat dismayed by the level of focus on the drug use in her life as opposed to other issues affecting her health. She said,

I’m an open person. I don’t mind…I had an appointment but I never came back because right now my main concern is not my drug use. It’s my environment and my health.,

while another patient cited the importance of being open to discussing his use due to its impact on his health. He explained,

If I don’t talk about it, it’s a secret I’m keeping still. If I don’t talk about it here or somewhere else, I get sicker. I gotta have a release, gotta be able to tell people something. And it teach me to be honest about what goes on with me, especially when it comes to medical.

Another patient felt it was important to discuss, but only when his use was at a more intense level. He said,

Very open, because I think not telling them could harm me more than any, but by me being able to speak freely about it, it kinda helps, because they can know how to handle it. I don’t bring it up if it’s not real bad, but if I go into, like, whereas I spend maybe $100.00 or something like that, then I think they should know.

Sixteen participants (52%) did not feel open discussing their use at all, often based on fear of their provider learning about their use and this knowledge somehow changing the care they were receiving. For example, patients were often worried about how discussing their drug use might affect their prescriptions, like this patient,

With Dr.______, I can talk to him about things but I’d rather not talk with him about my drug use ‘cause it’ll disturb my prescription.

Patients were also worried about being labeled a drug user or having their drug use behaviors recorded into their patient files, such as this participant,

Yeah, they always ask, but I always, like, lie, ‘cause I know it open up a lot of cans of worms and stuff where you be, you know, go in the file.

Other participants who were not open to discussing their drug use decided not to due to shame about their use or addictions. One participant said,

I never bring it up. It’s not personally something that I’m proud of. Actually, I’m ashamed of it.

and another shared,

Probably ashamed to some degree, although I do need to talk to my doctor about it. They know about all the other stuff, like the drinking and marijuana. I just never talked to them about crack cocaine.

Another patient simply avoided the conversation in order to avoid the anticipated messages he might receive from his physician regarding his use, saying,

I guess they would tell me how I shouldn’t be gettin’ high when I have this HIV, so that’s why I don’t feel like really talkin’ to ‘em.

This fear of receiving the message that a patient should not be using drugs was part of the reality of the medical visit, as a common theme in the interviews when patients were asked about the messages they receive about their drug use the most common was ‘stop or die.’

“Stop or Die”

In addition to asking participants how they feel about discussing their drug use, they were also asked what kind of messages they receive (if any) about their use from providers. Most participants shared that their physicians or nurse practitioners told them they needed to stop using. Very often this message was accompanied by a detailed description of how the drugs were affecting their health. For example, some providers gave detailed messages about how the drug use was affecting their organs in negative ways, as in the experience of this patient:

It’s not they fault I chose the wrong path, you know. I decided to use drugs, smoke crack, you know. They try to talk to me and tell me, “This is not good for you. It’s not helping your medication. It’s not helping your situation at all. This is doing this here to your organs and this to your heart and that to your brain.” I mean they sit down and talk to me. They let me know.

Another patient conveyed a similar approach, saying,

They understand what I’m doing, but what I’m doing is wrong because I’m hurting myself. I’m killing my organs. You know, they sympathize with me, but they get on me at the same time.

Providers also utilized threats with patients about the potential impact of their use on health and mobility. One patient explained,

I thought they were gonna take my leg off at one time, yeah, because I was shooting up in it and I had a huge abscess. And he was talking about taking it off, but I think that was to scare me, but it was a huge possibility too. Yeah, everybody’s just so nice.

The most prevalent message from providers was that continued drug use would lead to an early death, or from the patient perspective, “stop or die,” such as this participant,

Because they said if I ain’t stopped it’s gonna kill me.

Some providers echoed this sentiment, in more professional terms, citing the patients themselves as the actual barriers.

### Provider themes

#### Lack of training

Lack of preparedness emerged as the most prominent theme in the clinician results (*n* = 7). Common themes included a need for additional training and lack of familiarity with the drug using process and equipment. This was further exemplified with the presence of the props, which generated a myriad of questions about the injection process, the smoking process, and using naloxone for overdose reversal. This theme indicates that clinicians are not well-prepared to provide salient options for patients to reduce harm while using drugs. Quotes which illustrate this theme include:

I’ve never -now I would imagine this is probably something that they use to cook cocaine. This is probably saline in that, right? Is this a filter of some sort that they use? I talk to them mostly about sharing needles. I haven’t talked to them about the other parts of it. I do make sure they’re gonna see a substance abuse counselor and a couple of other things that I don’t have time to talk about or that I don’t know about (Nurse Practitioner).

In the above quote, the lack of familiarity with the other injection equipment would limit the options for harm reduction offered by the clinician, and may leave the patient at risk for Hepatitis transmission by sharing equipment such as cookers, or filters. For this clinician and others, additional education regarding drug use practices and harm reduction processes might result in less articulation of challenges and more readiness for implementation. In fact, most clinicians (*n* = 4) specifically cited a lack of education as an issue. They did not have the instruction in their clinical training necessary to provide Harm Reduction counseling or to engage in effective interviewing of patients. Other clinicians mentioned that they really needed more information related to drug use and Harm Reduction counseling, in the form of post-clinical training or updates to learn facts and build skills. Examples include:

Maybe four or five years ago we had a course where they actually came to give a workshop for the clinicians, talking about the different terms that are used, the different drugs that are used, and I think this is something that probably every two years or so we need to go through so that we’re aware of it…because a lot of times you don’t get that information in medical school, or a residency. Unless you have had a personal history or you have a close family member, you could be pretty naïve in terms of what’s going on. (Physician)

I just feel like we’re bad at this compared to how we treat heart disease or even HIV, that we’re so primitive in mental health and substance abuse. I also think that the new research, new information, you have to be seeking it out. You know, guidelines for blood pressure come at us bombarding us, but information about new medications that are being used for detox? I’m guessing that the majority of my colleagues don’t know about methadone use or buprenorphine…(Nurse Practitioner)

#### Discomfort with harm reduction

Many times, this lack of familiarity and experience in Harm Reduction seemed to create clinician discomfort, and other times, the discomfort seemed to emerge from attitudes related to the drug use, or drug users themselves. A strong theme emerged related to discomfort with drug use itself, which often translated into discomfort with patients using drugs, frustration with lack of change in use among patients, or concern that harm reduction methods would invite scrutiny. For example,

I guess it’s definitely dependent on the clinician’s comfort level, and if the clinician is ready to put on the knee high boots and get into the muck and mire of it…I think that might be a little bit more readily received from a peer than somebody from the ivory tower coming down, putting on the boots, and trying to show you or tell you. The patient already knows that this isn’t what they need to be doing, and they know that this is counterproductive to the promotion of their health. (Nurse Practitioner)

I do think it’s a way to be done, but I do think you’ll have a pushback from doctors. A lot of doctors are opposed to this type of counseling, cause people think it’s encouraging folks to use drugs, or encouraging deviant behavior, and you don’t wanna be seen as doing that…a lot of doctors are very concerned for this. Those are liberal views, talking about substance

abuse and sharing needles. (Physician)

#### Time and role

Another provider barrier was related to time and/or perceived role in the clinic. Some clinicians believed it was feasible to incorporate, while others shared that it was not their role or that time would inhibit the process. In each of these cases, it was the physicians who cited time-related barriers and role restrictions—feeling that this type of counseling should be provided by substance abuse counselors or other staff. This theme is probably influenced by the current structure of care in the United States, which often allocates less time in physician encounters than visits with nurse practitioners or other supportive clinic staff. Physicians explained:

My role, like I said for the most part, is at least just to try to help your CD4 and viral load status to the point where if you’re gonna do this, at least you’re not going to be able to transmit it. (Physician)

I think this is something that would be good for a group discussion…just because I think time wise I think during clinic, it would be very lengthy. They would all want to try it (the demonstration with equipment). (Physician)

For any integration to occur at the physician level, a paradigm shift in role may need to occur, as well as additional time for visits.

The challenges are really more of a time factor because you’re trying to do it during clinic hours when other patients are waiting to be seen. And it seems like the time flies when you’re in the room with a patient. I don’t know how people see patients in 10 min. (Physician)

The time barrier also seemed to be commonly linked to the frustration with other pressing responsibilities, such as paperwork, which interfere with providing more extensive clinical care services. This may be of particular concern in a publicly-funded clinic, whose paperwork requirements and grant writing are often completed by clinical leadership, and require time away from patients to complete.

Whether it’s a form to go to the dentist or whatever it is you need, we take care of it, so it isn’t the actual hands on with the patient. A lot of times, it’s the paperwork that takes you a little while to get done…Like 30 percent of the time I’m actually taking care of the patient, and 70 percent, I’m taking care of all the paperwork needs that they have. (Physician)

Despite the time barrier, the advanced practice nurses were very optimistic about incorporating Harm Reduction counseling into practice, although many agreed that physicians may not have the time to do so and that other clinic staff would need to be involved for successful implementation, as in these statements:

I think we’re doing a lot of it. There is more that we can do. I think using this kind of teaching tool would be really good, using a DVD would be good. I think it would have to have a certain class or a setting. Some of the things I could do, some of the tools, you know, a little thing here, a little thing there, because it would take up a lot of time in a regular visit, but I think it could be incorporated very easily. I think it would be really great, really, really great. (Nurse Practitioner)

I think it’s not only feasible, I think it should be. We actually do have the luxury of time so much more so than many of our colleagues in many settings. We can bring people back more often medically, when it’s really a mental health issue or a substance use issue. (Nurse Practitioner)

These quotes illustrate the potential for nurse practitioners to act as champions in moving implementation forward; however, patients have lingering concerns to address.

#### The patient as a barrier

Clinicians also cited patient-related barriers in their interviews, from getting them to show up for appointments, adhere to medications, or recognize the health problems that their use may cause. In these descriptions of challenges and barriers, clinicians often seemed suspicious of the motives of their own patients. Common challenges are illustrated in these quotes by different clinicians:

The biggest thing is people coming back for follow up, that’s the biggest challenge. Even with all of this stuff being put in place, if clients don’t come back to receive the services, it’s extremely hard, and the biggest carrot or the stick, the reason why they come back for primary care is ‘cause they got to have it in order to get those other services. If they want the housing or if they want the paperwork completed for whatever thing that they need, that’s why they’re coming in. (Nurse Practitioner)

I think the biggest challenge that I would see would be the drug use actually compromising their health, compromising their compliance with the medications, and increasing their risk for Hepatitis B, Hepatitis C, HIV, if they are not already impacted by it. I feel that with active drug use that is impacting their participation in safe sexual practices, so therefore not only impacting their health, but impacting the health of others in the community as well. (Physician)

#### Sex, drugs and rock & roll

Provider reports of how this challenge translated into discussion were more procedural. All seven providers in the study said that they address both sex and drug prevention in the context of the typical visit. Most of the time this discussion covered sexual prevention such as condoms, and how drug use might impact HIV medications or health. For example, providers said:

Sex, drugs and rock n’ roll is part of everybody’s appointment at all times, whether you’re using or not using.

We actually have, at the end of our progress note, the questions that are part of the prevention for positives protocol that actually goes through safe sexual practices, condom use, is your partner aware of your HIV status, avoiding drugs, alcohol, things that might impact on your sexual activity or the choices you make…

A common theme that providers often addressed and initiated in conversation with patients was the importance of disclosing their status to sexual partners. They explained:

Lately I’ve been talking with patients who have not disclosed their status to their partner, which is of great concern. Which is why perhaps the rates of HIV have not decreased to the way they should in heterosexual relationships.

I know that disclosing to people, I think that’s very important to tell them every single time, ‘you need to disclose your status if you’re having sex with anybody.’

In terms of assessing addiction, very few providers mentioned doing anything specific. Only one provider discussed using a screening tool related to assessing drug and alcohol use, a brief screening tool to assist a patient in determining whether their use may be problematic. This same provider was versed in the Transtheoretical model and Stages of Change, and seemed to already be incorporating a harm reduction approach to some degree, but she was the only provider to report this type of client-centered approach. She said:

I think that’s something that has to be repeated over and over and over. You’re assessing where they’re at, and so you just want to bring it up and then keep bringing it up. And then if they’re not interested in stopping, you can think of ways to minimize their chances of having problems and increasing the risk. So I just think it takes a lot of time. I think people will agree generally to a small change rather than disagree. I think they’re more apt to do something small…if I say stop drinking today, okay, that’s not gonna happen. But could they be more choosy about when they drink, or what they drink? Then I may have a chance.

## Discussion

### Summary of findings & implications for practice and policy

It is clear that the majority of patients could see the benefit of harm reduction interventions (safer injection counseling, safer stimulant use counseling, overdose prevention, and provision of equipment) as a routine part of the HIV care visit, and expressed enthusiasm about the possibility. This perspective was shared between injection drug users and those who smoked crack cocaine. This may indicate that for this small sample of clinics, harm reduction interventions would be well-received and valued by most patients who are actively using drugs. However, many patients expressed reluctance to discuss their drug use with providers, a certain barrier to meaningful integration of these interventions within the care visit. Perhaps the divide here between expressed optimism and reluctance to talk openly is explained by a social desirability bias (discussed further in limitations), or perhaps due to the perceived potential for harm reduction interventions to offer a more accepting approach that patients find appealing. While these questions might be answered with further research, they do indicate that provider approach matters, as does assessment of individual patient receptiveness. Also of note here is the divergent theme with a small subset of patients who were less receptive to the idea, with emphasis on two concerns: the potential stigma of being labeled a drug use by a provider, or the concern about a dramatic change in tone from their provider. While a harm reduction approach might engender a more accepting tone in the patient-provider relationship, it is clear that training must be provided to facilitate such a change. It is also important to consider these findings in relation to the existing literature in the US, which shows promise for successful integration of OST within HIV care (discussed above) albeit with slow uptake and continued unmet need. Perhaps the same barriers that interfere with wider diffusion of OST in HIV care are also present here.

Providers were indeed more focused on known barriers than patients, while maintaining cautious optimism about the possibility. While all providers seemed to understand the value of harm reduction interventions in mitigating harm in their patients’ lives, physicians cited more barriers and nurse practitioners expressed more optimism. The barriers cited by providers (physicians, in particular) included time, lack of knowledge, role-based barriers, and the patient as a barrier. Providers also reported some discomfort, and cited fears that they were not adequately prepared or knowledgeable enough to start a conversation about reducing harm. In addition, they exhibited a lack of familiarity with harm reduction equipment, which may have contributed to their discomfort in providing specific harm reduction interventions. Although clinicians, particularly the physicians, cited potential barriers to implementation, there is clear potential for Harm Reduction work to occur within this group. The findings indicate that nurse practitioners may be good local champions for diffusion, or to spearhead a pilot study on implementation of a Harm Reduction model in HIV care. It is possible that the difference between nurse practitioners and physicians is due to a difference in exposure to substance use and related issues in their course of study, or reflects the reality that nurse practitioners may have more time with each patient. However, it is unlikely that outside training in Harm Reduction prompted the difference, as 100% of physicians and 75% of NP/APNs in this study reported receiving it. Nevertheless, NPs/APNs cited fewer barriers to incorporating it into practice, and expressed openness or enthusiasm about the possibility. Yet, the number of NPs/APNs was incredibly small, so further examination of receptiveness of this group is certainly warranted.

A clear strategy to address most of the barriers identified in this study is to provide training. Training of providers might include incorporating a harm reduction philosophy and strategies to encourage patient disclosure of drug use, practical training around delivery of harm reduction interventions, treating addiction with opioid substitution therapy in the context of HIV care, and addressing drug-related stigma. A systematic review of efforts to change provider knowledge and attitudes related to addiction treatment indicated that participation in workshops facilitated changes in knowledge, attitudes, and even skill development (Walters et al., 2005), but ability to sustain such changes over time was not clear. Perhaps findings from this study can provide practical guidance on the content of trainings, or medical education curricula, starting on a local pilot basis and expanding nationally after such efforts have been properly evaluated. The national AIDS Education and Training Center (AETC) network is one promising resource which can relay these important skills into the training of HIV providers, in partnership with harm reduction organizations. Some AETCs have been implementing Harm Reduction training for more than a decade, although a more wide-scale, national approach would be expedient in diffusing this effort, capitalizing on expert AETC faculty in collaboration with harm reduction organizations. Partnership with a local harm reduction program may also assist in providing necessary equipment (safer injection kits, safer crack smoking kits, and naloxone for overdose prevention efforts). These partnerships can be identified and facilitated on a national level through engagement and discussion with the national AETC clinician training network and the Harm Reduction Coalition.

While lack of knowledge and discomfort can be addressed with comprehensive training, time and role-based barriers are system-level factors that require policy change to allow physicians in publicly-funded clinics more time with patients and proper billing for this time. This is an interesting time to consider policy-level change in this manner, as the Affordable Care Act and Medicaid expansion are underway, and likely to offer both opportunity and challenge related to system-level change. Yet, there are advocates working toward system-level change in HIV care and advocates working toward change in drug policy. Pooling or combining these forces may represent the best possibility to advocate for change in this arena to shift resources or allocate funding to harm reduction interventions within HIV care. Having drug policy advocates would be helpful in understanding how to convey the need for harm reduction funding in a climate where some aspects of the federal ban on funding for syringe exchange (as one example) continues.

Until such wide scale change occurs, motivated clinicians may choose to implement Harm Reduction counseling on an individual basis, serving as agents of change to help diffuse further into the care environment. Nurse practitioners may be well-suited for the role of champion, having a position paper delineating the role of the AIDS-certified nurse in areas of Harm Reduction/syringe exchange education, advocacy, treatment expansion, and support in curriculum development (Fisk, 1998). Despite a fifteen-year span since this piece was published, few strides have occurred and new champions must emerge for progress to occur. Thus, it is clear that these champions still work in an environment that is centered in abstinence-based approaches that further disenfranchise or deter the most vulnerable patients. Provider discomfort with harm reduction as it arose in this study is important in illustrating the difficulties that clinicians may face in implementing evidence-based policies that are not publicly supported, and in demonstrating the divide that often occurs between patient and provider when drug use is involved. Small, manageable steps toward integration of harm reduction interventions may offer promise to engage patients and build more successful relationships between provider and patient.

### Limitations & implications for research

There are several limitations to note in this research. First, although 100% of eligible providers in the clinics agreed to participate, the sample of *n* = 7 remains rather small. While the findings here illustrate the perceptions of those providers and may be transferable to similar clinic settings, they may not translate to a larger provider group. Further study is needed to expand to a more representative pool of providers, perhaps a survey-based study to examine the barriers that emerged in this study. Second, sampling was restricted to only three clinics, so the application of these findings should be in context with similar settings and patient populations. Third, both providers and patients understood that the purpose of the study was to discuss perceptions of harm reduction counseling in HIV care. Therefore, they may have felt pressured to provide socially desirable answers to questions pertaining to harm reduction receptiveness. However, the informed consent process was clear in articulating risks and benefits, and that participation in research would not affect patient care services at the clinic. Nevertheless, the openness of patients may be skewed if some participants feared the interview might affect their HIV care services. An additional limitation was the reliance on self-report data, although recall bias related to this limitation may be less applicable here since questions were focused on how participants felt currently, with the exception of learning about harm reduction in the past on the patient level.

Future research might begin with a larger survey, perhaps to a national sample of publicly-funded HIV clinics that serve injection and non-injection drug users, with the goal of confirming the exploratory findings here. Survey questions should be derived from or informed by the themes identified in this research, and should include further assessment of the magnitude and pervasiveness of barriers cited by the physician participants in this sample, as well as the openness and enthusiasm of advanced practice nurses. Similarly, patient attitudes may vary by geographic location and dependent on issues of perceived stigma, national or local drug policy, and the physician-patient relationship. Further exploration of a national patient sample of active drug users is necessary to confirm these findings, while smaller, qualitative studies in specific locations may be more useful in yielding descriptive findings regarding the extent of perceived stigma and local context by particular patient population.

An alternate and more actionable direction would be to use the data generated here to inform the development of training and implementation of harm reduction interventions (counseling plus provision of supplies) to be used in HIV clinic settings. The interventions could then be tested for efficacy in a small pilot study in these three Chicago clinics, and slowly expanded to other settings with appropriate cultural tailoring for the patient population. While the findings here may encourage the development of pilot interventions centered in publicly-funded, Chicago-based clinics in low-income, African-American communities, the ultimate research goal would include a larger-scale feasibility study in a variety of HIV care venues, in the hopes of diffusing effective interventions nationally.

Other research priorities should include studies addressing special populations not covered in this study who experience more intense stigma and punishment with active drug use, such as pregnant women or adolescents. Similarly, studying the potential for harm reduction integration in alternative venues would be useful, particularly where drug-related harm and overdose risk should be mitigated, such as in methadone clinics, drug treatment systems, and correctional facilities. Finally, research to further understand the lack of uptake of OST in HIV clinics in the US, or intervention studies that examine ways to increase uptake, are also needed—to further explore ideas for meeting the needs of PWIDs.

## Conclusions

A harm reduction approach involves recognizing and respecting the slow nature of behavior change, assessing each individual patient where they are, providing a myriad of options to reduce harm, and creating a mutually-beneficial, nonjudgmental environment that facilitates open communication. The potential to incorporate specific harm reduction interventions in publicly-funded HIV clinics was explored here, with cautiously optimistic results. With few exceptions, patients were receptive to harm reduction counseling and supplies being provided by their clinicians, even while not always comfortable discussing their drug use. Those who expressed reluctance indicated that approach would be critical in maintaining the concerned tone normally conveyed by clinicians in the usual visit. Providers were mixed, with more barriers cited by physicians, and more enthusiasm expressed by nurse practitioners. The creation of a pilot training program that clearly addresses cited barriers seems a logical implication for practice within these particular clinics or in similar settings. A larger study is also warranted to confirm these exploratory findings in a more representative sample. Nevertheless, the findings reveal some potential to conquer the divide between the often-marginalized patient and their provider, by illustrating some receptiveness to harm reduction interventions in HIV clinics. The evidence-base for harm reduction methods, the scarcity of harm reduction services, the receptiveness of patients, and the enthusiasm of nurse practitioners are compelling reasons to move forward.

## Supplemental Information

10.7717/peerj.1932/supp-1 Supplemental Information 1 Supplementary File; Kappa interpretation huidelines & scores

10.7717/peerj.1932/supp-2 Supplemental Information 2 Consolidated criteria for reporting qualitative research—COREQ checklist

## Additional Information and Declarations

### Data Availability

The following information was supplied regarding data availability: Raw data may not be provided due to the extensive material within that may identify individual participants and clinics.
