# Supplementary material for: Harm reduction interventions in HIV care: a qualitative exploration of patient and provider perspectives
Source: PeerJ. 2016 Apr 14;4:e1932. doi: 10.7717/peerj.1932 (PMC4841234; doi:10.7717/peerj.1932)
Supplement: Supplemental Information 1 [file peerj-04-1932-s001.docx]

| **Kappa** | **Interpretation** |
| --- | --- |
| < 0 | No agreement |
| 0.0 – 0.20 | Slight agreement |
| 0.21 – 0.40 | Fair agreement |
| 0.41 – 0.60 | Moderate agreement |
| 0.61 – 0.80 | Substantial agreement |
| 0.81 – 1.00 | Almost perfect agreement |

**Cohen’s Kappa Scores**

| **Code** | **# of Observed Agreements** | **# of Agreements Expected by Chance** | **Cohen’s Kappa** | **Confidence Interval** | **Strength of Agreement** |
| --- | --- | --- | --- | --- | --- |
| Separation from Other | 142  (99.30%) | 125.3 (87.59%) | 0.944 | 0.834 – 1.054 | Almost Perfect |
| Abuse History | 143  (100%) | 135.2  (94.56%) | 1.000 | 1.000 – 1.000 | Almost Perfect |
| Lack of Trust Users | 142  (99.30%) | 134.3  (93.90%) | 0.885 | 0.661 – 1.109 | Almost Perfect |
| Gender Dynamics | 142  (99.30%) | 130.6  (91.32%) | 0.919 | 0.762 – 1.077 | Almost Perfect |
| Drug History | 143  (100%) | 131.5  (91.96%) | 1.000 | 1.000 – 1.000 | Almost Perfect |
| Parenting Drugs | 143  (100%) | 131.5  (91.96%) | 1.000 | 1.000 – 1.000 | Almost Perfect |
| Self HR | 138  (96.50%) | 136.1  (95.16%) | 0.277 | -0.345 – 0.900 | Fair Agreement |
| HR Learn | 140  (97.90%) | 138.1  (96.54%) | 0.393 | -0.399 – 1.194 | Fair Agreement |
| Stay Safe | 142  (99.30%) | 138.1  (96.56%) | 0.797 | 0.399 – 1.194 | Substantial Agreement |
| Prep | 143  (100%) | 135.2  (94.56%) | 1.000 | 1.000 – 1.000 | Almost Perfect |
| Stressors | 143  (100%) | 135.2  (94.56%) | 1.000 | 1.000 – 1.000 | Almost Perfect |
| Living with HIV | 143  (100%) | 137.1  (95.89%) | 1.000 | 1.000 – 1.000 | Almost Perfect |
| Hustle | 142  (99.30%) | 140  (97.92%) | 0.664 | 0.006 – 1.321 | Substantial Agreement |
| OD History | 143  (100%) | 139.1  (97.24%) | 1.000 | 1.000 – 1.000 | Almost Perfect |
| Stigma | 141  (98.60%) | 137.1  (95.88%) | 0.660 | 0.193 – 1.128 | Substantial Agreement |
| Sex Life | 143  (100%) | 137.1  (95.89%) | 1.000 | 1.000 – 1.000 | Almost Perfect |
| Change Environment | 142  (99.30%) | 140  (97.92%) | 0.664 | 0.006 – 1.321 | Substantial Agreement |
| Diagnosis | 143  (100%) | 137.1  (95.89%) | 1.000 | 1.000 – 1.000 | Almost Perfect |
| Entry into Treatment | 143  (100%) | 135.2  (94.56%) | 1.000 | 1.000 – 1.000 | Almost Perfect |
| Relationship with Provider | 143  (100%) | 133.3  (93.25%) | 1.000 | 1.000 – 1.000 | Almost Perfect |
| Feelings about Clinic | 143  (100%) | 135.2  (94.56%) | 1.000 | 1.000 – 1.000 | Almost Perfect |
| Disc Drug Use Clinic | 143  (100%) | 135.2  (94.56%) | 1.000 | 1.000 – 1.000 | Almost Perfect |
| HR Crack | 143  (100%) | 139.1  (97.24%) | 1.000 | 1.000 – 1.000 | Almost Perfect |
| HR Trust | 143  (100%) | 139.1  (97.24%) | 1.000 | 1.000 – 1.000 | Almost Perfect |
| 12 Step | 143  (100%) | 139.1  (97.24%) | 1.000 | 1.000 – 1.000 | Almost Perfect |
| Ideal Visit | 143  (100%) | 141.0  (98.61%) | 1.000 | 1.000 – 1.000 | Almost Perfect |
