# Supplementary material for: Harm reduction interventions in HIV care: a qualitative exploration of patient and provider perspectives
Source: PeerJ. 2016 Apr 14;4:e1932. doi: 10.7717/peerj.1932 (PMC4841234; doi:10.7717/peerj.1932)
Supplement: Supplemental Information 2 [file peerj-04-1932-s002.pdf]

Consolidated criteria for reporting qualitative research (COREQ) checklist

Harm Reduction in HIV Care: A qualitative exploration of patient and provider perspectives

Suzanne Carlberg-Racich, PhD, MSPH

Domain 1: Research team and reflexivity
Personal Characteristics
1. Interviewer/facilitator
Which author/s conducted the interview or focus group? **Suzanne Carlberg-Racich**
2. Credentials
What were the researcher's credentials? E.g. PhD, MD **At the time of the study, MSPH and PhD Candidate – this manuscript is derived from a doctoral dissertation.**
3. Occupation
What was their occupation at the time of the study? **Training Specialist at the Midwest AIDS Training and Education Center, Chicago, University of Illinois at Chicago**
4. Gender
Was the researcher male or female? **Female**
5. Experience and training
What experience or training did the researcher have? **Work on a collaborative qualitative study with similar methodology, plus a few doctoral-level courses in qualitative analysis and methods.**

Relationship with participants
6. Relationship established
Was a relationship established prior to study commencement? **There was an established relationship with clinical leadership at the clinic sites for study buy-in, but not the patient participants.**
7. Participant knowledge of the interviewer
What did the participants know about the researcher? **e.g. personal goals, reasons for doing the research – Clinic sites were informed that the project was part of fulfilling a doctoral dissertation. Individual participants were informed of the purpose of the study (per informed consent requirements established by the IRB).**
8. Interviewer characteristics
What characteristics were reported about the interviewer/facilitator? e.g. Bias, assumptions, reasons and interests in the research topic **There is mention of hiring an independent coder to mitigate any bias from the PI's harm reduction experience, but this can be expanded if desired.**

Domain 2: study design
Theoretical framework
9. Methodological orientation and Theory
What methodological orientation was stated to underpin the study? e.g. grounded theory, discourse analysis, ethnography, phenomenology, content analysis **Phenomenology**

Participant selection
10. Sampling
How were participants selected? e.g. purposive, convenience, consecutive, snowball **Purposive**
11. Method of approach
How were participants approached? e.g. face-to-face, telephone, mail, email **Flyers in the clinics with tear-off**

tabs including the researcher's phone number; also, referral from care providers.
12. Sample size
How many participants were in the study? **N = 38**
13. Non-participation
How many people refused to participate or dropped out? Reasons? **No one refused participation or dropped out. One person who expressed interest did not meet the inclusion criteria.**

Setting
14. Setting of data collection
Where was the data collected? e.g. home, clinic, workplace **Clinic and university office (for two participants who wanted to keep their participation anonymous from the clinic staff)**
15. Presence of non-participants
Was anyone else present besides the participants and researchers? **No. Private space was used for all interviews.**
16. Description of sample
What are the important characteristics of the sample? e.g. demographic data, date **The most important characteristics were: living with HIV/AIDS, actively using heroin, cocaine/crack, and being a patient of one of three chosen publicly-funded HIV clinics. All patient participants were African-American. Age and Level of education are provided as well (in table form in the manuscript). Provider participants had one important characteristic: currently providing care in the chosen clinics, at the mid-level provider level or higher (non-prescribing providers were not included). Research shows that patients respond differently to advice from providers at the mid-level and MD levels, so they were the focus.**

Data collection
17. Interview guide
Were questions, prompts, guides provided by the authors? Was it pilot tested? **Yes, the interview guide was developed by the author, and pilot tested.**
18. Repeat interviews
Were repeat interviews carried out? If yes, how many? **No repeat interviews were needed.**
19. Audio/visual recording
Did the research use audio or visual recording to collect the data? **Yes, audio recording occurred with participant permission.**
20. Field notes
Were field notes made during and/or after the interview or focus group? **Only minimal note taking occurred to focus on maximizing eye contact and interaction with participants.**
21. Duration
What was the duration of the interviews or focus group? **Approximately an hour, with some minor variation when participants were particularly brief or verbose.**
22. Data saturation
Was data saturation discussed? **Yes.**
23. Transcripts returned
Were transcripts returned to participants for comment and/or correction? **Transcripts were not returned, but the researcher engaged participants in member-checking immediately following interviews.**

Domain 3: analysis and findings

Data analysis

24. Number of data coders
How many data coders coded the data? **2**
25. Description of the coding tree
Did authors provide a description of the coding tree? **The coding process is explained – from the development of index codes (driven from the research questions, interview guide, and concept map) to grounded codes that emerged naturally from the data.**
26. Derivation of themes
Were themes identified in advance or derived from the data? **Index codes were identified in advance based on the questions, and additional codes emerged while coding, but themes emerged naturally from the data.**
27. Software
What software, if applicable, was used to manage the data? **AtlasTi**
28. Participant checking
Did participants provide feedback on the findings? **Provider participants gave feedback on the findings when results were reported back to the clinics. Patient participants were not approached a second time, but member-checking was done post-interview, and member-checking of themes was completed with community members that fit the inclusion criteria for the study. The researcher did not have easy access to the patients a second time.**

Reporting
29. Quotations presented
Were participant quotations presented to illustrate the themes / findings? Was each quotation identified? e.g. participant number **Quotes were identified with basic descriptors to ensure anonymity (gender and drug of choice only).**
30. Data and findings consistent
Was there consistency between the data presented and the findings? **Yes**
31. Clarity of major themes
Were major themes clearly presented in the findings? **Yes**
32. Clarity of minor themes
Is there a description of diverse cases or discussion of minor themes? **Yes**

# Human Studies
Please provide an empty copy of the human participant consent form you used as a confidential Supplemental File here <https://peerj.com/manuscripts/7559/files/>. **Done**
